# Quantum Non-Markovian Environment-to-System Backflows of Information: Nonoperational vs. Operational Approaches

**DOI:** 10.3390/e24050649

**Published:** 2022-05-05

**Authors:** Adrián A. Budini

**Affiliations:** 1 Consejo Nacional de Investigaciones Científicas y Técnicas (CONICET), Centro Atómico Bariloche, Avenida E. Bustillo Km 9.5, Bariloche 8400, Argentina; 2 Universidad Tecnológica Nacional (UTN-FRBA), Fanny Newbery 111, Bariloche 8400, Argentina

**Keywords:** open quantum systems, quantum non-Markovianity

## Abstract

Quantum memory effects can be qualitatively understood as a consequence of an environment-to-system backflow of information. Here, we analyze and compare how this concept is interpreted and implemented in different approaches to quantum non-Markovianity. We study a nonoperational approach, defined by the distinguishability between two system states characterized by different initial conditions, and an operational approach, which is defined by the correlation between different outcomes associated to successive measurement processes performed over the system of interest. The differences, limitations, and vantages of each approach are characterized in detail by considering diverse system–environment models and dynamics. As a specific example, we study a non-Markovian depolarizing map induced by the interaction of the system of interest with an environment characterized by incoherent and coherent self-dynamics.

## 1. Introduction

The time-evolution of both classical and quantum systems may develop memory effects [1,2,3,4]. Nevertheless, the characterization and definition of these effects is quite different in both regimes [5,6,7]. As is well known, in a classical regime memory effects can be rigorously defined in a probabilistic approach. The independence or dependence of conditional probabilities on the previous system history define, respectively, the (memoryless) Markovian and non-Markovian regimes [1].

In a quantum regime, one is immediately confronted with an extra aspect. In fact, the state of a quantum system (and consequently its history) can only be determined by performing a measurement process, which intrinsically implies a perturbation to its (originally unperturbed) dynamics. Therefore, the definition of memory effects and quantum non-Markovianity can be tackled from two intrinsically different approaches. In *nonoperational approaches*, memory effects are defined by taking solely into account the properties of the unperturbed open system dynamics (its propagator). In *operational approaches*, memory effects are defined by the statistical properties of different outcomes associated to system measurement processes and transformations (such as unitary ones).

A wide variety of measures and memory witnesses have been utilized in the context of nonoperational approaches (see reviews [5,6,7]). The first proposals correspond to deviations of the system propagator from divisibility [8,9] and a nonmonotonous behavior of the trace distance (TD) between two distinct system states [10,11]. In this context, memory effects were associated to an *environment-to-system backflow of information*: information stored in the initial system state is transferred to the environmental degrees of freedom; their influence on the system at later times implies a backflow of information that leads to memory effects. In spite of this clear and well-motivated interpretation [12,13,14], the precise assessment of this concept is still under debate [15,16,17,18,19,20,21,22,23].

The basic idea of operational approaches is to appeal to the standard definition of memory effects in terms of probabilities [1]. Hence, the (quantum) system must be subjected to a set of measurement processes such that their statistical properties determine the presence or absence of memory effects [24,25,26,27]. The study and understanding of this approach was performed in the recent literature [28,29,30,31,32,33,34], including alternative definitions and analysis of information flows [35,36].

The main goal of this paper is to analyze and to compare how the concept of environment-to-system backflow of information is interpreted and implemented in operational and nonoperational approaches. As a nonoperational memory witness, we take the TD between two different systems’ initial states [10,11], also taking into account the bounds on its revival behavior that have been characterized recently [22,23]. As an operational memory witness, we consider a conditional past–future (CPF) correlation [26,27], both in deterministic and random schemes [36]. The comparison is performed by considering different system–environment models and analyzing in each case the information flows from the two perspectives. We consider statistical mixtures of Markovian system evolutions and systems coupled to incoherent [16] and coherent casual bystander environments [37], which are characterized by a self-dynamics that is independent of the system degrees of freedom. In addition, we consider (standard) unitary system–environment models [2]. As a specific model, we study a depolarizing map induced by the interaction of a system with a finite set of incoherent degrees of freedom. In this regime, as well as in a quantum coherent one, we explain how and why both approaches lead to different notions of quantum non-Markovianity and environment-to-system backflows of information.

The paper is outlined as follows. In Section 2 we review the definition and main properties of the considered nonoperational [10,11,22,23] and operational [26,27,36] approaches. In Section 3 we study both approaches by considering different system–environment models. In Section 4, we study the depolarizing map. In Section 5, we provide the conclusions.

## 2. Quantum Non-Markovianity

Here, we briefly review the main characteristics of the different approaches to quantum non-Markovianity.

### 2.1. Nonoperational Approach

If the open system is not affected or perturbed during its evolution, the unique object that allows defining the presence or absence of memory effects is its (unperturbed) density matrix propagator. The rigorous theory of quantum dynamical semigroups [38] motivate associating the (memoryless) quantum Markovian regime with propagators whose time-evolution obey a Lindblad equation (or Gorini–Kossakowski–Sudarshan–Lindblad equation). Consequently, any (scalar) measure or property that quantifies departures of the system propagator from a Lindblad equation can be taken as a witness of quantum memory effects.

Lindblad equations lead to completely positive propagators between two arbitrary times [38]. As is well known, completely positive transformations lead to very specific contractive properties for different distance measures and entropic quantities [39]. For example, the TD between two arbitrary density matrixes ρ and σ, defined as D(ρ,σ)≡(1/2)Tr|ρ−σ|, under a completely positive transformation Φ, fulfills the inequality D(Φ[ρ],Φ[σ])≤D(ρ,σ). Consequently, it is possible to *define* quantum Markovianity by the condition [10,11]

(1) $$D\left(\rho_{t+\tau }^{s},\sigma_{t+\tau }^{s}\right)\leq D\left(\rho_{t}^{s},\sigma_{t}^{s}\right),$$

where $\rho_{t}^{s}$ and $\sigma_{t}^{s}$ are two arbitrary evolved system states that differ in their initial conditions, $\rho_{0}^{s}\neq \sigma_{0}^{s}.$ Alternatively, one can interpret that quantum memory effects are present whenever this inequality is not fulfilled for a set of two arbitrary time intervals t≥0 and τ>0.

In spite of the simplicity and efficacy of the previous theoretical frame, in general, it is not possible to know or infer which physical processes are involved when the contractive condition (1) is not fulfilled. A remarkable advance in this direction was recently obtained in Refs. [22,23] by establishing the inequality

(2) $$\begin{array}{ccc} D(\rho_{t+\tau }^{s},\sigma_{t+\tau }^{s}) & \leq & D\left(\rho_{t}^{s},\sigma_{t}^{s}\right)+D\left(\rho_{t}^{e},\sigma_{t}^{e}\right)\\ & & \;\;\;\;\;+D\left(\rho_{t}^{se},\rho_{t}^{s}⊗\rho_{t}^{e}\right)+D\left(\sigma_{t}^{se},\sigma_{t}^{s}⊗\sigma_{t}^{e}\right). \end{array}$$

Here, $\rho_{t}^{se}$ and $\sigma_{t}^{se}$ are the evolved system–environment states with initial conditions $\rho_{0}^{se}=\rho_{0}^{s}⊗\rho_{0}^{e}$ and $\sigma_{0}^{se}=\sigma_{0}^{s}⊗\sigma_{0}^{e}.$ As usual, the system and bath states follow from partial trace operations, $\rho_{t}^{s}={\mathrm{Tr}}_{e}\left[\rho_{t}^{se}\right]$ and $\rho_{t}^{e}={\mathrm{Tr}}_{s}\left[\rho_{t}^{se}\right].$ The asymmetry between system and environment (s↮e) is introduced by taking in both cases the same initial environmental state, $\rho_{0}^{e}=\sigma_{0}^{e}.$

The result (2) only relies on the triangle inequality fulfilled by the TD. Thus, it is valid for arbitrary system–environment models. In addition, this expression allows to bounding the environment-to-system backflow of information *defined* by the “revivals”

(3) $$D\left(\rho_{t+\tau }^{s},\sigma_{t+\tau }^{s}\right)-D\left(\rho_{t}^{s},\sigma_{t}^{s}\right)>0.$$

The remaining (bounding) contributions in the rhs of Equation (2) have a *clear physical interpretation*. One can relate the contribution $D(\rho_{t}^{e},\sigma_{t}^{e})$ to changes in the environmental state, while the terms $D\left(\rho_{t}^{se},\rho_{t}^{s}⊗\rho_{t}^{e}\right)+D\left(\sigma_{t}^{se},\sigma_{t}^{s}⊗\sigma_{t}^{e}\right)$ measure the correlations established between the system and the environment [22,23]. Nevertheless, it is important to realize that these physical processes do not guarantee the developing of revivals. The right conclusion is that *given* that there exists revivals, their origin can related to changes in the environmental state or to the establishing of system–environment correlations.

It was also proven that the inequality (2) remains valid when the TD is replaced by a telescopic relative entropy and the square root of a quantum Jensen–Shannon divergence [22,23]. Thus, the interpretation of the bounds remains the same when using these entropic quantities.

### 2.2. Operational Approach

In a probabilistic frame, given a sequence of system states x→y→z with joint probability P(z,y,x), Markovianity is defined by the condition

(4) P(z,y,x)=P(z|y)P(y|x)P(x),

where P(b|a) denotes in general the conditional probability of *b* given a. By Bayes rule, the equality (4) implies the (memoryless) condition P(z|y,x)=P(z|y). Similar constraints emerge when considering higher joint probabilities involving an arbitrary number of events [1].

For quantum systems, the definition of Markovianity in terms of probabilities unavoidably implies performing a set of system measurement processes. In Refs. [24,25], by means of a process tensor formalism, the Markovian condition is taken into account for arbitrary (higher order) joint probabilities. Nevertheless, for *quantum* systems coupled to standard environment models (standard classical noises and/or unitary system–environment interaction models), only three measurement events are enough for detecting departures from a (probabilistic) Markovian regime [26,27]. In such a case, the condition (4) can be conveniently rewritten as a CPF independence,

(5) P(z,x|y)=P(z|y)P(x|y).

This result follows straightforwardly by using P(z,x|y)=P(z,y,x)/P(y), where $P\left(y\right)=\sum_{z,x}P\left(z,y,x\right).$

The CPF independence (5) implies that any (conditional) correlation between past and future events witnesses memory effects. Correspondingly, a CPF correlation is defined as [26,27]

(6) $$C_{pf}{\left(t,\tau \right)|}_{\overset{˘}{y}}\overset{d/r}{=}\sum_{z,x}zx\left[P\left(z,x|\overset{˘}{y}\right)-P\left(z|\overset{˘}{y}\right)P\left(x|\overset{˘}{y}\right)\right],$$

where {x} and {z} are the (past and future) measurement outcomes. The time dependence (t,τ) emerges because the past, present, and future measurements are performed at the initial time t=0, at time t, and t+τ, respectively. Evidently, $C_{pf}{\left(t,\tau \right)|}_{\overset{˘}{y}}$ vanishes in a (probabilistic) Markovian regime (Equation (5)).

In Equation (6), the change $y\to \overset{˘}{y}$ was introduced, which is stretchy related with the definition of memory effects and information flows in this approach. Two different measurement schemes are necessary [36]. In a deterministic scheme (denoted with the supra d), after the intermediate measurement (whose outcome defines the conditional property) no change is introduced. Hence, $\overset{˘}{y}=y.$ In a random scheme (denoted with the supra r), after the intermediate measurement, the system state is randomly chosen $\left(y\to \overset{˘}{y}\right)$ over the set of possible states associated to the outcomes {y}. The CPF correlation is defined with this renewed conditional state.

In the deterministic scheme, the CPF correlation $\left[C_{pf}\left(t,\tau \right){|}_{\overset{˘}{y}}\overset{d}{\neq }0\right]$ detects memory effects (departures with respect to Equation (4), or equivalently, Equation (5)) independently of the specific system–environment model. In the random scheme, a nonvanishing CPF correlation $[C_{pf}\left(t,\tau \right){|}_{\overset{˘}{y}}\overset{r}{\neq }0],$ by *definition,* detects the presence of environment-to-system backflows of information (or bidirectional system–environment information flows). This relation is motivated by the complementary case $C_{pf}{\left(t,\tau \right)|}_{\overset{˘}{y}}\overset{r}{=}0$ that applies when the environment (which induces the memory effects $C_{pf}\left(t,\tau \right){|}_{\overset{˘}{y}}\overset{d}{\neq }0)$ is unperturbed by its coupling with the system [36].

The previous characteristics of the deterministic and random schemes can be easily understood from the properties of projective measurements performed over bipartite systems [37]. Interestingly, the formalism remains the same and is also valid for purely (classically) incoherent system–environment arrangements.

### 2.3. Bipartite Propagator vs. Single Propagator

Before comparing both approaches (next section), here, we clarify which dynamical objects determine each one. In the nonoperational approach, the presence of memory effects (TD revivals defined by Equation (3)) can be determined after knowing solely the system (single) propagator. In contrast, for determining the bound defined by Equation (2), it is necessary to know the bipartite system–environment propagator specified for a given initial bath state.

In contrast, the operational approach can only be characterized by knowing (exact or approximate) the bipartite propagator for different initial bath states (the initial one and the bath state after the intermediate measurement). As a matter of fact, the CPF correlation (6) can be written as a function of the joint probability $P(z,\overset{˘}{y},x).$ Assuming that the three measurements are projective ones, in the deterministic scheme it reads [36]

(7) $$\frac{P(z,\overset{˘}{y},x)}{P(x)}\overset{d}{=}{\mathrm{Tr}}_{se}\left(E_{z}G_{t+\tau ,t}^{se}\left[\rho_{\overset{˘}{y}}⊗{\mathrm{Tr}}_{s}\left(E_{\overset{˘}{y}}G_{t,0}^{se}\left[\rho_{x}^{se}\right]\right)\right]\right),$$

while in the random scheme it is [36]

(8) $$\frac{P(z,\overset{˘}{y},x)}{P(x)}\overset{r}{=}{\mathrm{Tr}}_{se}\left(E_{z}G_{t+\tau ,t}^{se}\left[\rho_{\overset{˘}{y}}⊗{\mathrm{Tr}}_{s}\left(G_{t,0}^{se}\left[\rho_{x}^{se}\right]\right)\right]\right)℘\left(\overset{˘}{y}|x\right).$$

In these expressions, $G_{t+\tau ,t}^{se}$ is the *bipartite propagator* between *t* and t+τ. In addition, $E_{m}\equiv \left|m\rangle \langle m\right|$ and $\rho_{m}\equiv \left|m\rangle \langle m\right|$$\left[m=z,\overset{˘}{y},x\right]$ represent the (positive) effect measurement operators and postmeasurement states, respectively. The sets {|m〉}$\left[m=z,\overset{˘}{y},x\right]$ are the eigenstates of each measured observable. Furthermore, $\rho_{x}^{se}\equiv \rho_{x}⊗\rho_{0}^{e}$ and $P\left(x\right)=\langle x|\rho_{0}^{s}|x\rangle .$ The random scheme is parameterized by an arbitrary conditional probability $℘(\overset{˘}{y}|x)$ that defines the change in the system state $\left(y\to \overset{˘}{y}\right)$ after the intermediate measurement.

The different dependence of both approaches on the bipartite propagator leads to strong different conclusions about memory effects and information flows, which are analyzed in the next section.

## 3. Comparing Both Approaches

In order to perform a systematic comparison we consider different system–environment models and approximations. In general, we assume that the bipartite system–environment state $\rho_{t}^{se}$ evolves as

(9) $$\frac{d}{dt}\rho_{t}^{se}=\left(L_{s}+L_{e}+L_{se}\right)\left[\rho_{t}^{se}\right],$$

where $L_{s}$ and $L_{e}$ define the self-dynamics of the system and the environment, respectively, while $L_{se}$ defines their mutual interaction. This interaction term may be unitary or include dissipative couplings.

### 3.1. Born–Markov Approximation

For systems weakly coupled to their environments, the Born–Markov approximation [2] allows to write the bipartite state as

(10) $$\rho_{t}^{se}≃\rho_{t}^{s}⊗\rho_{0}^{e},$$

where $\rho_{t}^{s}$ is the system state, while $\rho_{0}^{e}$ is the (almost) unperturbed environment state.

When this approximation is valid, in the *nonoperational approach*, it is simple to check that Equation (2) reduces to Equation (1). In fact, $D\left(\rho_{t}^{e},\sigma_{t}^{e}\right)=D\left(\rho_{t}^{se},\rho_{t}^{s}⊗\rho_{t}^{e}\right)=D\left(\sigma_{t}^{se},\sigma_{t}^{s}⊗\sigma_{t}^{e}\right)=0.$ Furthermore, $\rho_{t}^{s}$ can be well approximated by a Lindblad equation, which guarantees the absence of any revival in $D(\rho_{t}^{s},\sigma_{t}^{s}).$ Thus, the dynamics is Markovian.

In the *operational approach*, by introducing the approximation (10) into Equations (7) and (8) straightforwardly, it follows that $C_{pf}{\left(t,\tau \right)|}_{\overset{˘}{y}}\overset{d/r}{=}0$ (Equation (6)). These results are independent of which observables are measured. Thus, the dynamics is Markovian.

In this case (Equation (10)), both approaches coincide. Strong differences appear in the cases studied below.

### 3.2. Casual Bystander Environments

A wide class of “non-Markovian” dynamics can be derived by assuming that the system interacts with a “casual bystander” environment. These baths are defined by the independence of their marginal states $\rho_{t}^{e}={\mathrm{Tr}}_{s}\left[\rho_{t}^{se}\right]$ of any degree of freedom of the system. Alternatively, the time evolution of $\rho_{t}^{e}$ can be written in the environment Hilbert space without involving any operator or state of the system. These properties must be valid for arbitrary system and environment (separable) initial conditions.

For fulfilling the previous properties, the interaction term $L_{se}$ in the general evolution (9) must be restricted such that

(11) $${\mathrm{Tr}}_{s}\left(L_{se}\left[\rho_{t}^{se}\right]\right)=A\left[\rho_{t}^{e}\right],$$

where A is an arbitrary superoperator acting on $\rho_{t}^{e}$ that does not have any dependence on the system degrees of freedom. In general, this constraint can only be satisfied by dissipative (nonunitary) system–environment couplings. On the other hand, the bath dynamics can be quantum [37] or a classical (incoherent) one [16].

In the *nonoperational approach*, the independence of the environment state on the system degrees of freedom cannot be translated to any restriction on the inequality defined by Equation (2). In fact, under the constraint (11), the TD may or not present revivals, *property that can only be cheeked for each specific model*. Thus, some dynamics are classified as Markovian and other as non-Markovian. The unique simplification that can be introduced is to assume that the environment state does not evolve in time, $\rho_{t}^{e}=\rho_{0}^{e},$ that is, the environment begins in its stationary state. In this case, Equation (2) reduces to

(12) $$\begin{array}{ccc} D\left(\rho_{t+\tau }^{s},\sigma_{t+\tau }^{s}\right)-D\left(\rho_{t}^{s},\sigma_{t}^{s}\right) & \leq & D(\rho_{t}^{se},\rho_{t}^{s}⊗\rho_{0}^{e})\\ & & \;\;\;\;\;+D(\sigma_{t}^{se},\sigma_{t}^{s}⊗\rho_{0}^{e}). \end{array}$$

Even in this case $(\rho_{t}^{e}=\rho_{0}^{e}),$ the TD may or may not present revivals, that is, depending on the model, the system may be classified as Markovian or non-Markovian.

In Equation (12), any environment-to-system backflow of information can be related to the establishing of the correlations $D\left(\rho_{t}^{se},\rho_{t}^{s}⊗\rho_{0}^{e}\right)+D\left(\sigma_{t}^{se},\sigma_{t}^{s}⊗\rho_{0}^{e}\right).$ Certainly, the system–environment correlations (always) changes in time. Nevertheless, even when there are no revivals in the TD system–environment, correlations are established. This feature represents a central problem for the interpretation of this approach. In addition, here, the environment state is completely independent of the system (and even of time). Thus, the revivals of the TD must be taken as a (mathematical) model-dependent property whose origin cannot be related to any physical process that implies a *physical* transfer of information from the environment to the system.

A different perspective emerges in the *operational approach*. By using the independence of the environment state $\left[\rho_{t}^{e}={\mathrm{Tr}}_{s}\left(\rho_{t}^{se}\right)\right]$ of any degree of freedom of the system, it is possible to check that the joint probability (7) of the deterministic scheme *does not fulfill* the Markov property (4). In contrast, it is simple to check that the joint probability (8) of the random scheme *fulfills* the Markov property (4). Consequently, a casual bystander environment leads to the CPF correlations (Equation (6))

(13) $$C_{pf}\left(t,\tau \right){{|}_{\overset{˘}{y}}\overset{d}{\neq }0,\;\;\;\;\;\;\;\;\;\;C_{pf}\left(t,\tau \right)|}_{\overset{˘}{y}}\overset{r}{=}0.$$

In this approach, the property $C_{pf}{\left(t,\tau \right)|}_{\overset{˘}{y}}\overset{d}{\neq }0,$ valid for any model under the constraint (11), implies that the system dynamics is non-Markovian. Its origin can be related to the establishing of (arbitrary) system–environment correlations. On the other hand, the property $C_{pf}{\left(t,\tau \right)|}_{\overset{˘}{y}}\overset{r}{=}0,$ which is valid for arbitrary measurement processes and specific models, is read as the *absence* of bidirectional system–environment information flows. In fact, given that the environment is characterized by a self-dynamics that is completely independent of the system, any environment-to-system backflow of information (as detected in the nonoperational approach) does not rely on any physical process that affects the environment state or its dynamics.

The meaning of the previous analysis is clarified by specifying different bipartite models that fulfill the evolution (9) and the constraint (11).

#### 3.2.1. Classical Mixture of Quantum Markovian Dynamics

Given a set of *different* system Lindblad superoperators $\{L_{s}^{c}\},$ which may include both unitary and dissipative contributions, and given a set of normalized positive weights $\{p_{c}\},$$\sum_{c}p_{c}=1,$ a classical statistical mixture of Markovian dynamics is defined by the bipartite state

(14) $$\rho_{t}^{se}=\sum_{c}\exp\left(tL_{s}^{c}\right)\left[\rho_{0}^{s}\right]⊗p_{c}|c\rangle \langle c|.$$

Here, {|c〉〈c|} is a set of projectors associated to the environment space. The marginal system and environment states read

(15) $$\rho_{t}^{s}=\sum_{c}p_{c}\exp\left(tL_{s}^{c}\right)\left[\rho_{0}^{s}\right],\;\;\;\;\;\;\rho_{t}^{e}=\sum_{c}p_{c}|c\rangle \langle c|.$$

Memory effects in this kind of non-Markovian system dynamics have been explored in the literature [40,41,42,43,44]. Notice that the environment does not have any dynamics. Even more, the system dynamics can be performed by mixing in a random way (with weight $p_{c}$) each of the evolved Markovian system states $\exp\left(tL_{s}^{c}\right)\left[\rho_{0}^{s}\right].$ Thus, the detection of an environment-to-system backflow of information via Equation (3) seems to have a formal mathematical interpretation rather than a physical one. On the other hand, in the operational approach, this case is characterized by Equation (13), which guaranties the presence of memory effects $C_{pf}{\left(t,\tau \right)|}_{\overset{˘}{y}}\overset{d}{\neq }0$ but not any bidirectional information flow, $C_{pf}{\left(t,\tau \right)|}_{\overset{˘}{y}}\overset{r}{=}0.$

#### 3.2.2. Interaction with Stochastic Classical Degrees of Freedom

When the system interacts with stochastic classical degrees of freedom, the bipartite state can be written as

(16) $$\rho_{t}^{se}=\sum_{c}\rho_{t}^{c}⊗p_{c}\left(t\right)|c\rangle \langle c|.$$

In contrast to the previous case (Equation (14)), the weights $\left\{p_{c}\left(t\right)\right\}$ are time-dependent and the evolution of the states $\left\{\rho_{t}^{c}\right\}$ may involve coupling between all of them. In fact, under the constraint (11), the more general evolution can be written as [16]

(17) $$\frac{d{\tilde{\rho }}_{t}^{c}}{dt}=L_{s}^{c}\left[{\tilde{\rho }}_{t}^{c}\right]-\sum_{c^{\prime }}\gamma_{c^{\prime }c}{\tilde{\rho }}_{t}^{c}+\sum_{c^{\prime }}\gamma_{cc^{\prime }}S_{cc^{\prime }}\left[{\tilde{\rho }}_{t}^{c^{\prime }}\right].$$

Here, ${\tilde{\rho }}_{t}^{c}\equiv p_{c}\left(t\right)\rho_{t}^{c}.$ Thus, $p_{c}\left(t\right)={\mathrm{Tr}}_{s}\left({\tilde{\rho }}_{t}^{c}\right).$ Furthermore, $\left\{S_{cc^{\prime }}\right\}$ are arbitrarily completely positive system transformations, which are trace preserving ${\mathrm{Tr}}_{s}\left(S_{cc^{\prime }}\left[\rho \right]\right)={\mathrm{Tr}}_{s}\left(\rho \right).$ Consequently, the environment probabilities $\left\{p_{c}\left(t\right)\right\}$ obey a classical master equation

(18) $$\frac{dp_{c}\left(t\right)}{dt}=-\sum_{c^{\prime }}\gamma_{c^{\prime }c}p_{c}\left(t\right)+\sum_{c^{\prime }}\gamma_{cc^{\prime }}p_{c^{\prime }}\left(t\right),$$

which in turn shows the role played by the coupling rates $\{\gamma_{c^{\prime }c}\}.$ In contrast, the system dynamics depart from a Markovian (Lindblad) evolution. From some specific models, it is possible to recover some phenomenological non-Markovian master equations (see, for example, [45,46,47]).

In the nonoperational approach, it is very difficult to predict if a given dynamics (Equation (16)) leads or not to revivals in the TD. If the incoherent degrees of freedom begin in their stationary state, $p_{c}\left(0\right)=\lim_{t\to \infty }p_{c}\left(t\right),$ one is confronted with the bounds defined by Equation (12). Even in this case, one cannot predict when there exists or not an environment-to-system backflow of information.

Interestingly, the origin of the contributions $D\left(\rho_{t}^{se},\rho_{t}^{s}⊗\rho_{0}^{e}\right)+D\left(\sigma_{t}^{se},\sigma_{t}^{s}⊗\rho_{0}^{e}\right)$ in Equation (12) (or in general in Equation (2)) can be easily read from the evolution (17). In fact, this equation shows that the system evolution is *totally conditioned* to the environment dynamics. The contributions $L_{s}^{c}$ are “active” whenever the environment is in the state |c〉〈c|. Furthermore, the system suffers the transformation $\rho \to S_{c^{\prime }c}\left[\rho \right]$ whenever the environment “jumps” between the states $c\to c^{\prime }.$ This is the physical mechanism that leads to the system–environment correlations, which in turn does not imply any system-dependent change in the environment state or dynamics. Thus, the interpretation of revivals in the TD as environment-to-system backflow of information is again controversial.

Independently of the Lindblad contributions $\{L_{s}^{c}\},$ the superoperators $\{S_{cc^{\prime }}\},$ and rates $\{\gamma_{c^{\prime }c}\},$ the operational approach is characterized by Equation (13), that is, the dynamics is non-Markovian $\left[C_{pf}\left(t,\tau \right){|}_{\overset{˘}{y}}\overset{d}{\neq }0\right]$ without the development of any bidirectional system–environment information flow $[C_{pf}\left(t,\tau \right){|}_{\overset{˘}{y}}\overset{r}{=}0].$

#### 3.2.3. Environmental Quantum Degrees of Freedom

The condition Equation (11) can be satisfied even when the environment is a quantum one, that is, it develops coherent behaviors. In this case, the bipartite state can be written as

(19) $$\rho_{t}^{se}=\sum \rho_{t}^{c}⊗p_{c}\left(t\right)|c_{t}\rangle \langle c_{t}|.$$

In contrast to Equation (16), due to the quantum nature of the environment, the projectors $\{|c_{t}\rangle \langle c_{t}|\}$ are time-dependent. In fact, they define the base in which the environment density matrix $\rho_{t}^{e}$ is diagonal. The more general bipartite evolution (9) under the constraint (11), in its diagonal representation, is given by [37]

(20) $$\begin{array}{ccc} \frac{d}{dt}\rho_{t}^{se} & = & \left(L_{s}+L_{e}\right)\left[\rho_{t}^{se}\right]+\sum_{\alpha }\Gamma_{\alpha }\;B_{\alpha }S_{\alpha }\left[\rho_{t}^{se}\right]B_{\alpha }^{†}\\ & & -\frac{1}{2}\sum_{\alpha }\Gamma_{\alpha }{\left\{B_{\alpha }^{†}B_{\alpha },\rho_{t}^{se}\right\}}_{+}, \end{array}$$

where ${\left\{\cdot ,\cdot \right\}}_{+}$ is an anticommutator operation. Furthermore, $\left\{B_{\alpha }\right\}$ are arbitrary environment operators, while $S_{\alpha }$ are completely positive trace-preserving system superoperators. The rates $\left\{\Gamma_{\alpha }\right\}$ set the environment dynamics. In fact,

(21) $$\frac{d}{dt}\rho_{t}^{e}=L_{e}\left[\rho_{t}^{e}\right]+\sum_{\alpha }\Gamma_{\alpha }\;\left(B_{\alpha }\rho_{t}^{e}B_{\alpha }^{†}-\frac{1}{2}{\left\{B_{\alpha }^{†}B_{\alpha },\rho_{t}^{e}\right\}}_{+}\right),$$

which is a Lindblad dynamics completely independent of the system degrees of freedom. These evolutions recover, as particular cases, some phenomenological collisional models introduced in the literature (see, for example, [48,49,50]).

The physical interpretation of the evolution (20) is quite similar to that of Equation (17). In fact, here, the application of the system superoperators $S_{\alpha }$ occurs whenever the environment suffers a transition associated to the operators $B_{\alpha }.$ This (unidirectional) mechanism defines how the system–environment correlations are built up.

In the nonoperational approach, even when the environment begins in its stationary state $\rho_{0}^{e}=\lim_{t\to \infty }\rho_{t}^{e}$ (where $\rho_{t}^{e}$ obeys Equation (21)), it is not possible to infer for an arbitrary model the presence or absence of revivals in the TD (Equation (3)). In contrast, the operational approach is still characterized by Equation (13).

### 3.3. Unitary System–Environment Interactions

Independently of the specific models, the correlation between the system and the casual bystander environments introduced previously does not involve quantum entanglement [51] (see the separable states Equations (14), (16) and (19)). In contrast, quantum entanglement may emerge when considering Hamiltonian (time-reversible) system–environment interactions. In fact, solely for special system–environment initial conditions, a bipartite unitary dynamics does not induce quantum entanglement [52,53,54].

The total Hamiltonian is written as

(22) $$H_{T}=H_{s}+H_{e}+H_{I}.$$

Each contribution corresponds to the system, environment, and interaction Hamiltonians, respectively. The bipartite propagator is

(23) $$G_{t,t_{0}}^{se}\left[•\right]=\exp\left[-i\left(t-t_{0}\right)H_{T}\right]•\exp\left[+i\left(t-t_{0}\right)H_{T}\right].$$

In the *nonoperational approach*, each contribution in the rhs of Equation (2) makes complete sense in this context. In fact, almost all unitary interactions lead to a change in the environment state and also induce the development of (arbitrary) system–environment correlations. When revivals in the TD develop, Equation (2) defines a bound with a clear physical meaning. Nevertheless, in general, it is not possible to infer which kind of dynamics develop or do not develop revivals in the TD. Even for a given (Hamiltonian) model, depending on the underlying parameters, the system dynamics may be Markovian or not. Consequently, it is not clear which physical property defines the boundary between Markovian and non-Markovian dynamics.

In the *operational approach*, given that the state and dynamics of the environment are in general modified by a unitary interaction, instead of Equation (13), here, it follows

(24) $$C_{pf}\left(t,\tau \right){{|}_{\overset{˘}{y}}\overset{d}{\neq }0,\;\;\;\;\;\;\;\;\;\;C_{pf}\left(t,\tau \right)|}_{\overset{˘}{y}}\overset{r}{\neq }0.$$

Both inequalities can be supported by performing a perturbation theory based on projector techniques [31]. Consistently, it has been shown that even close to the validity of a Born–Markov approximation, the operational approach can detect memory effects [34].

The inequality $C_{pf}{\left(t,\tau \right)|}_{\overset{˘}{y}}\overset{d}{\neq }0$ implies that the system dynamics is non-Markovian (system–environment correlations are developed during the evolution), while $C_{pf}{\left(t,\tau \right)|}_{\overset{˘}{y}}\overset{r}{\neq }0$ detects the presence of bidirectional information flows. In fact, here, the environment state and evolution always depend on the system degrees of freedom.

There exists a unique exception to Equation (24), which reduces to Equation (13). Hence, even when the environment state is modified, for any system observables, one obtains $C_{pf}{\left(t,\tau \right)|}_{\overset{˘}{y}}\overset{r}{=}0.$ While this property is certainly *undesirable*, this case has a clear physical interpretation. It emerges when, in a given environmental base {|e〉}, the diagonal part of the bipartite propagator (23) can be written as

(25) $$\langle e|G_{t,0}^{se}\left[•\right]|e\rangle =T_{t,0}^{\left(e\right)}\langle e|•|e\rangle ,$$

where $T_{t,0}^{\left(e\right)}$ is a *system* (density matrix) propagator that parametrically depends on each environmental state {|e〉}. The condition (25) is fulfilled, *for example*, when the environment and interaction Hamiltonians commutate

(26) $$[H_{e},H_{I}]=0.$$

Introducing the condition (25) into Equations (7) and (8), it is possible to check that $C_{pf}{\left(t,\tau \right)|}_{\overset{˘}{y}}\overset{d}{\neq }0,$ and $C_{pf}{\left(t,\tau \right)|}_{\overset{˘}{y}}\overset{r}{=}0.$ This last equality *does not imply* that the environment in not affected. It emerges because the system state assumes the structure

(27) $$\rho_{t}^{s}={\mathrm{Tr}}_{e}\left(G_{t,0}^{se}\left[\rho_{0}^{s}⊗\rho_{0}^{e}\right]\right)=\sum_{e}\langle e|\rho_{0}^{e}|e\rangle T_{t,0}^{\left(e\right)}\left[\rho_{0}^{s}\right].$$

Therefore, the system evolution can be written as a statistical superposition of unitary maps, quite similar to Equation (15). Consequently, for unitary system–environment models, the condition $C_{pf}{\left(t,\tau \right)|}_{\overset{˘}{y}}\overset{r}{=}0$ allows to detect when the system dynamics (even between measurements) can be represented by a *Hamiltonian ensemble*, a property that has been of interest in the recent literature [55].

## 4. Example

In this section, we consider an explicit example of the dynamics discussed previously. The quantum system (s), taken for simplicity as a two-level system, interacts with an incoherent environment (e) (see Section 3.2.2), which here is defined by four discrete states, denoted as {|1〉,|2〉,|3〉,|4〉}. Correspondingly, the bipartite system–environment state is written as

(28) $$\rho_{t}^{se}=\sum_{k=1,2,3,4}{\tilde{\rho }}_{k}\left(t\right)⊗|k\rangle \langle k|.$$

The system and environment states then read

(29) $$\rho_{t}^{s}=\sum_{k=1,2,3,4}{\tilde{\rho }}_{k}\left(t\right),\;\;\;\;\;\;\;\rho_{t}^{e}=\sum_{k=1,2,3,4}p_{k}\left(t\right)|k\rangle \langle k|,$$

where $p_{k}\left(t\right)={\mathrm{Tr}}_{s}\left[{\tilde{\rho }}_{k}\left(t\right)\right].$ The evolution of the unnormalized system states ${\left\{{\tilde{\rho }}_{k}\left(t\right)\right\}}_{k=1}^{k=4}$ is taken as

(30a) $$\begin{array}{ccc} \frac{d{\tilde{\rho }}_{4}\left(t\right)}{dt} & = & -\gamma {\tilde{\rho }}_{4}\left(t\right)+\phi \sum_{k=1,2,3}\sigma_{k}{\tilde{\rho }}_{k}\left(t\right)\sigma_{k}, \end{array}$$

(30b) $$\begin{array}{ccc} \frac{d{\tilde{\rho }}_{k}\left(t\right)}{dt} & = & -\phi {\tilde{\rho }}_{k}\left(t\right)+\left(\frac{\gamma }{3}\right)\sigma_{k}{\tilde{\rho }}_{4}\left(t\right)\sigma_{k},\;\;\;k=1,2,3.\;\;\;\;\;\;\;\; \end{array}$$

In this expression, γ and ϕ are characteristic coupling rates. Furthermore, the set of Pauli matrixes is denoted as $\left(\sigma_{x},\sigma_{y},\sigma_{z},I\right)↔\left(\sigma_{1},\sigma_{2},\sigma_{3},\sigma_{4}\right),$ where I is the identity matrix in the two-dimensional system Hilbert space. From Equations (30a) and (30b), the evolution of the environment populations is defined by the following classical master equation

(31a) $$\begin{array}{ccc} \frac{dp_{4}\left(t\right)}{dt} & = & -\gamma p_{4}\left(t\right)+\phi \sum_{k=1,2,3}p_{k}\left(t\right), \end{array}$$

(31b) $$\begin{array}{ccc} \frac{dp_{k}\left(t\right)}{dt} & = & -\phi p_{k}\left(t\right)+\left(\frac{\gamma }{3}\right)p_{4}\left(t\right),\;\;\;k=1,2,3.\; \end{array}$$

This equation is completely independent of the system degrees of freedom. Thus, the evolutions (30a) and (30b) has a simple interpretation. When the environment suffers the transition $|4\rangle \overset{\gamma /3}{\to }|k\rangle$ or the transition $|k\rangle \overset{\phi }{\to }|4\rangle$(k=1,2,3), the transformation $\sigma_{k}•\sigma_{k}$ is *conditionally* applied over the open quantum system.

Equations (30a) and (30b) can be solved after specifying the bipartite initial conditions. We consider a separable state, $\rho_{0}^{se}=\rho_{0}^{s}⊗\rho_{0}^{e},$ which implies ${\tilde{\rho }}_{k}\left(0\right)=\rho_{0}^{s}p_{k}\left(0\right).$ In general, each auxiliary state ${\tilde{\rho }}_{k}\left(t\right)$ can be written as a superposition of the Pauli channels acting on the initial system state $\rho_{0}^{s},$ that is,

(32) $${\tilde{\rho }}_{k}\left(t\right)=\sum_{j=1,2,3,4}g_{k}^{j}\left(t\right)\sigma_{j}\rho_{0}^{s}\sigma_{j},$$

where $\left\{g_{k}^{j}\left(t\right)\right\}$ are (sixteen) scalar functions that depend on time. Their initial conditions are $g_{k}^{4}\left(0\right)=p_{k}\left(0\right)$ and $g_{k}^{j}\left(0\right)=0,$ with j=1,2,3, and k=1,2,3,4. The evolution of the set $\left\{g_{k}^{j}\left(t\right)\right\}$ follows after inserting the previous expression for ${\tilde{\rho }}_{k}\left(t\right)$ into Equations (30a) and (30b). Consistent with their definition, $p_{k}\left(t\right)={\mathrm{Tr}}_{s}\left[{\tilde{\rho }}_{k}\left(t\right)\right],$ the environment populations are recovered as

(33) $$p_{k}\left(t\right)=\sum_{j=1,2,3,4}g_{k}^{j}\left(t\right).$$

### 4.1. Depolarizing Dynamics

The evolution of the auxiliary states Equations (30a) and (30b) is (structurally) the same for the states $\{{\tilde{\rho }}_{1}\left(t\right),{\tilde{\rho }}_{2}\left(t\right),{\tilde{\rho }}_{3}\left(t\right)\}.$ Thus, if we consider environment initial conditions where $p_{1}\left(0\right)=p_{2}\left(0\right)=p_{3}\left(0\right),$ from Equations (29) and (32), it follows that the solution map $\rho_{0}^{s}\to \rho_{t}^{s}$ must be a depolarizing channel [39], that is,

(34) $$\rho_{t}^{s}=w\left(t\right)\rho_{0}^{s}+\frac{1-w(t)}{3}\sum_{k=1,2,3}\sigma_{k}\rho_{0}^{s}\sigma_{k},$$

where the positive weight w(t), from Equation (32), follows as

(35) $$w\left(t\right)=\sum_{k=1,2,3,4}g_{k}^{4}\left(t\right).$$

Consistently, $\left[1-w\left(t\right)\right]/3=\sum_{k=1,2,3,4}g_{k}^{j}\left(t\right),$ with j=1,2,3.

The more natural initial conditions for the environment are their stationary populations $p_{k}^{\infty }\equiv \lim_{t\to \infty }p_{k}\left(t\right),$ where $p_{k}\left(t\right)$ is defined by Equations (31a) and (31b). Straightforwardly, we obtain

(36) $$p_{4}^{\infty }=\frac{\phi }{\gamma +\phi },\;\;\;\;\;\;\;\;p_{k}^{\infty }=\frac{1}{3}\frac{\gamma }{\gamma +\phi }\;\;\;\;\;\left(k=1,2,3\right).$$

Under the assumption $p_{k}\left(0\right)=p_{k}^{\infty },$ after obtaining the set $\left\{g_{k}^{j}\left(t\right)\right\}$ in an explicit analytical way, the function w(t) that characterizes the depolarizing channel Equation (34) can be written as

(37) $$w\left(t\right)=\frac{\left(\gamma^{2}+3\phi^{2}\right)}{3{\left(\gamma +\phi \right)}^{2}}+\frac{4\gamma \phi }{3{\left(\gamma +\phi \right)}^{2}}e^{-(\gamma +\phi )t}+\frac{2\gamma }{3(\gamma +\phi )}e^{-\phi t},$$

which consistently satisfies w(0)=1. Furthermore, $\lim{}_{t\to \infty }w\left(t\right)\neq 0.$ On the hand, the environment dynamics is stationary, that is, $p_{k}\left(t\right)=p_{k}\left(0\right)=p_{k}^{\infty }$ (Equation (36)).

### 4.2. Operational vs. Nonoperational Quantum Non-Markovianity

In the *nonoperational approach*, quantum non-Markovianity is defined by the revivals in the trace distance between two different initial states, Equation (3). By using that $\left(I/2\right)=\left(\rho +\sum_{k=1,2,3}\sigma_{k}\rho \sigma_{k}\right)/4$ [39], the depolarizing map (34) can be rewritten as $\rho_{t}^{s}=w\left(t\right)\rho_{0}^{s}+\left(1/3\right)\left[1-w\left(t\right)\right]\left(2I-\rho_{0}^{s}\right).$ Thus, the trace distance straightforwardly can be written as

(38) $$D\left[\rho_{t}^{s},\sigma_{t}^{s}\right]=\left|\frac{4w(t)-1}{3}\right|D\left[\rho_{0}^{s},\sigma_{0}^{s}\right]\equiv d\left(t\right)D\left[\rho_{0}^{s},\sigma_{0}^{s}\right]$$

where $D[\rho_{0}^{s},\sigma_{0}^{s}]$ is the trace distance between the two initial states $\rho_{0}^{s}$ and $\sigma_{0}^{s}.$ Notice that the decay of the trace distance does not depend on the initial states, being dictated by the function d(t).

**Figure 1:**
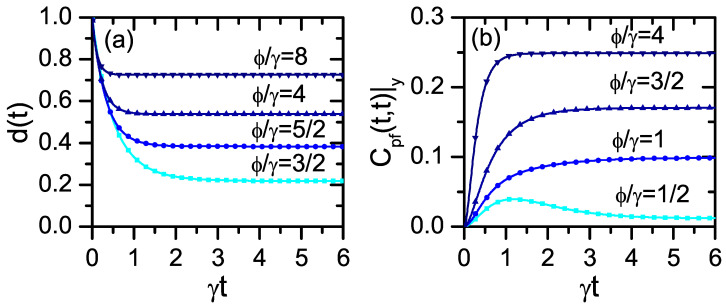
(**a**) Decay of the trace distance d(t) (Equation (38)) corresponding to the models (30a) and (30b). (**b**) Time dependence of the CPF correlation $C_{pf}{\left(t,t\right)|}_{\overset{˘}{y}}$ in the deterministic scheme [56] corresponding to the same model. The value of the quotient ϕ/γ is indicated in each plot.

In Figure 1a, we plot the function d(t) for different values of the characteristic parameter ϕ/γ. As expected from Equation (37), $D[\rho_{t}^{s},\sigma_{t}^{s}]$ decays in a monotonous way without developing any revival. Thus, under the trace distance criteria, the dynamics is *Markovian*, and there is not any environment-to-system backflow of information. Nevertheless, notice that for any value of ϕ/γ, system–environment correlations are built up during the dynamics [see Equation (28)]. This feature, which is irrelevant for the TD decay behavior, is relevant for the CPF correlation.

In the *operational approach*, the presence of memory effects is witnessed by the CPF correlation (Equation (6)) in the deterministic scheme. We assume that the three measurements are projective ones, all of them being performed in the *z*-direction of the Bloch sphere. Furthermore, the initial condition of the system is taken as $\rho_{0}^{s}=|\psi \rangle \langle \psi |,$ where |ψ〉 is an eigenstate of the *x*-Pauli matrix. Explicit general expression for $C_{pf}{\left(t,\tau \right)|}_{\overset{˘}{y}}$ in terms of the coefficients $\left\{g_{k}^{j}\left(t\right)\right\}$ can be found in Ref. [57] (see corresponding Appendix D). Under the previous assumptions, the CPF correlation can be obtained in an analytical way, which is written in [56]. Simple expressions are obtained for specific values of the decay rates. For example, for ϕ=γ, it follows

(39) $$\begin{array}{ccc} & & C_{pf}{\left(t,\tau \right)|}_{\overset{˘}{y}}\overset{d}{=}\frac{4}{81}\left(1-e^{-\gamma t}\right)\left(1-e^{-\gamma \tau }\right)\\ & & \;\;\;\;\;\;\;\;\;\;\;\;\;\;\;\;\;\;\times (2+e^{-\gamma t}+e^{-\gamma \tau }+5e^{-\gamma (t+\tau )}). \end{array}$$

Due to the symmetry of the problem, in all cases $C_{pf}{\left(t,\tau \right)|}_{\overset{˘}{y}}$ does not depend on the value of the conditional $\overset{˘}{y}=\pm 1.$

In Figure 1b, we plot the CPF correlation at equal times $C_{pf}{\left(t,t\right)|}_{\overset{˘}{y}}$ for different values of ϕ/γ. In contrast to the nonoperational approach, here, for all possible values of the characteristic parameter ϕ/γ it is fulfilled $C_{pf}{\left(t,\tau \right)|}_{\overset{˘}{y}}\overset{d}{\neq }0,$ which indicates a *non-Markovian* regime. In fact, the system is strongly correlated with the environment (Equation (28)).

The system–environment correlations emerge due to a unidirectional dependence of the system dynamics on the environment transitions (Equations (30a) and (30b)). In fact, the environment populations do not depend on the system degrees of freedom (see Equations (31a) and (31b)). These properties are relevant in the random scheme and imply that $C_{pf}{\left(t,\tau \right)|}_{\overset{˘}{y}}\overset{r}{=}0$ (Equation (13)). This result is valid for arbitrary measurement processes, indicating in the operational approach the absence of any environment-to-system backflow of information.

### 4.3. Environment-to-System Backflow of Information

In the previous section, we concluded that both approaches differ in the classification of the dynamics (Markovian vs. non-Markovian), but (due to different reasons) agree in the absence of any environment-to-system backflow of information. Here, we show that in general, both approaches also differ in this last aspect. Different mechanisms can be proposed for obtaining a revival in the trace distance Equation (38).

#### 4.3.1. Slow Modulation of the Stationary Environment State

First, we consider the same model (Equations (30a) and (30b)), but in addition, it is assumed that the characteristic rates are time-dependent, γ→γ(t),ϕ→ϕ(t), with

(40) γ(t)=γ[1+b(t)]>0,ϕ(t)=ϕ[1−b(t)]>0.

Here, b(t) is an arbitrary function of time that fulfills the constraint −1<b(t)<1. The previous structure is chosen for simplifying the argument and calculus. Nevertheless, we remark that similar dependences can be implemented in different experimental situations (see for example Ref. [58]). The more relevant aspect is that the assumption (40) can be implemented by affecting solely the environmental degrees of freedom (see Equations (31a) and (31b)).

In addition, in Equation (40), it is assumed that

(41) $$\left|\frac{d}{dt}b\left(t\right)\right|\ll \gamma ,\;\;\;\;\left|\frac{d}{dt}b\left(t\right)\right|\ll \phi .$$

Hence, the time dependence of b(t) can be considered slow with respect to the decay times (1/γ) and (1/ϕ). Consequently, the full dynamics can be described in an adiabatic approximation, where the full bipartite system in the long time regime (γt≫1,ϕt≫1) rapidly adjusts to the instantaneous values of γ(t) and ϕ(t). In particular, in this regime, the environment populations from Equation (36) can be written as

(42) $$p_{4}^{\infty }\left(t\right)≃\frac{\phi }{\gamma +\phi }\left[1-b\left(t\right)\right],\;\;\;\;p_{k}^{\infty }\left(t\right)≃\frac{1}{3}\frac{\gamma }{\gamma +\phi }\left[1+b\left(t\right)\right],$$

where k=1,2,3. For simplicity, we assumed that (γ−ϕ)≪(γ+ϕ), which allows to approximate γ(t)+ϕ(t)=(γ+ϕ)+b(t)(γ−ϕ)≃(γ+ϕ).

In the long time regime, the *nonoperational approach* is characterized by the value $\lim_{t\to \infty }w\left(t\right)\neq 0$ (see Equations (37) and (38)). For time-independent rates, this quantity can be written in terms of the stationary populations ${\left\{p_{k}^{\infty }\right\}}_{k=1}^{k=4}$ (Equation (36)) as $\lim_{t\to \infty }w\left(t\right)={\left[p_{4}^{\infty }\right]}^{2}+\sum_{k=1,2,3}{\left[p_{k}^{\infty }\right]}^{2}.$ Given that in the slow modulation regime (Equation (41)) these values become time dependent, $p_{k}^{\infty }⟶p_{k}^{\infty }\left(t\right)$ (Equation (42)), it follows that

(43) $$w\left(t\right)\overset{slow}{≃}{\left[p_{4}^{\infty }\left(t\right)\right]}^{2}+\sum_{k=1,2,3}{\left[p_{k}^{\infty }\left(t\right)\right]}^{2},\;\;\;\;\gamma t\gg 1,\;\phi t\gg 1.$$

Therefore, under the previous hypothesis, the stationary values of the TD in Figure 1a $\left[d\left(t\right)=\left|4w(t)-1\right|/3\right]$ become proportional to the arbitrary function b(t). This result implies that one can obtain *arbitrary revivals in the trace distance* (Equation (38)) by choosing different time dependences of the function b(t). Alternatively, an *arbitrary environment-to-system backflow of information can be produced* by changing solely in a slow way the (“stationary”) environment populations. Nevertheless, we remark that the full dynamics is essentially the same as in the static-rate case. While one can associate the revivals in the TD to the system–environment correlations, these correlations have the same origin and structure as in the absence of revivals, such as in Figure 1a (static case) and when b(t) does not lead to revivals.

In the deterministic scheme, the *operational approach* is characterized by the stationary value [56]

(44) $$\lim_{\begin{array}{c} t\to \infty \\ \tau \to \infty \end{array}}C_{pf}{\left(t,\tau \right)|}_{\overset{˘}{y}}\overset{d}{=}\frac{8\gamma {\left(\gamma -3\phi \right)}^{2}\left(\gamma +3\phi \right)}{81{\left(\gamma +\phi \right)}^{4}},$$

which can also be written in terms of ${\left\{p_{k}^{\infty }\right\}}_{k=1}^{k=4}$ (Equation (36)). Thus, under the same conditions that guarantee the slow modulation regime (Equations (41) and (42)), the stationary values of $C_{pf}{\left(t,t\right)|}_{\overset{˘}{y}}$ plotted in Figure 1b also become proportional to the function b(t). Nevertheless, in this approach, this property does not imply the presence of any backflow of information. In fact, given that the environment state does not depend at all on the system degrees of freedom, even in the slow modulation regime, it follows that $C_{pf}{\left(t,\tau \right)|}_{\overset{˘}{y}}\overset{r}{=}0$ (Equation (13)). In this way, it is clear that both the nonoperational and operational approaches also strongly disagree in this aspect.

#### 4.3.2. Quantum Coherent Contributions in the Environment Dynamics

The system–environment dynamics associated to the depolarizing channel (Equations (30a) and (30b)) can alternatively be represented through a Lindblad equation. In fact, the evolution of the bipartite state $\rho_{t}^{se}$ can be written as

(45) $$\begin{array}{ccc} \frac{d\rho_{t}^{se}}{dt} & = & +\frac{\gamma }{3}\sum_{k=1,2,3}\left(B_{k}\sigma_{k}\left[\rho_{t}^{se}\right]\sigma_{k}B_{k}^{†}-\frac{1}{2}{\left\{B_{k}^{†}B_{k},\rho_{t}^{se}\right\}}_{+}\right)\\ & & +\phi \sum_{k=1,2,3}\left(B_{k}^{†}\sigma_{k}\left[\rho_{t}^{se}\right]\sigma_{k}B_{k}-\frac{1}{2}{\left\{B_{k}B_{k}^{†},\rho_{t}^{se}\right\}}_{+}\right)\\ & & -i[H_{e},\rho_{t}^{se}], \end{array}$$

where the bath operators are $B_{k}\equiv |k\rangle \langle 4|,$k=1,2,3. As before, {|1〉,|2〉,|3〉,|4〉} are the environment base. Defining the states ${\tilde{\rho }}_{k}\equiv \langle k|\rho_{t}^{se}|k\rangle ,$ it is simple to check that the first two lines of the previous Lindblad dynamics recover the time evolution introduced in Equations (30a) and (30b).

From Equation (45), it is simple to check that the bath state $\left(\rho_{t}^{e}={\mathrm{Tr}}_{s}\left[\rho_{t}^{se}\right]\right)$ obeys a Lindblad equation that, even with the extra contribution $-i[H_{e},\rho_{t}^{se}],$ is independent of the system degrees of freedom. Thus, the environment is still a casual bystander one (see Equations (20) and (21)). In order to obtain a (system) depolarizing channel (Equation (34)), the symmetry between the bath states {|1〉,|2〉,|3〉} must be granted. For example, the Hamiltonian

(46) $$H_{e}=\frac{\Omega }{2}\sum_{k=1,2,3}(|k\rangle \langle 4|+|4\rangle \langle k|)$$

fulfills this property.

In consistence with the solution defined by Equations (28) and (32), here, the bipartite state is written as

(47) $$\rho_{t}^{se}=\sum_{k=1,2,3,4}\left(\sigma_{k}\rho_{0}^{s}\sigma_{k}\right)⊗\varrho_{t}^{k},$$

where $\left\{\varrho_{t}^{k}\right\}$ are states in the environment Hilbert space. In order to obtain analytically treatable solutions, we assume the bipartite initial condition

(48) $$\rho_{0}^{se}=\rho_{0}^{s}⊗\rho_{0}^{e}=\rho_{0}^{s}⊗|4\rangle \langle 4|.$$

Under this assumption $(\rho_{0}^{e}=|4\rangle \langle 4|),$ given that the underlying system stochastic dynamics associated to Equation (45) is the same as in the incoherent case (Equations (30a) and (30b)), it follows that the system state goes back to the initial condition $\rho_{0}^{s}$ whenever the environment goes back to the state |4〉. This property straightforwardly follows from $\sigma_{k}^{2}=I.$ Therefore, under the assumption (48), here, the depolarizing map Equation (34) is defined with the function

(49) $$w\left(t\right)={\mathrm{Tr}}_{e}\left[\varrho_{t}^{4}\right]=\langle 4|\rho_{t}^{e}|4\rangle ,$$

where $\rho_{t}^{e}$ is the density matrix of the environment. Consistently, $\left[1-w\left(t\right)\right]/3={\mathrm{Tr}}_{e}\left[\varrho_{t}^{k}\right]=\langle k|\rho_{t}^{e}|k\rangle ,$ with k=1,2,3. Consequently, the decay of the trace distance is proportional to the bath population $\langle 4|\rho_{t}^{e}|4\rangle .$ Its explicit analytical expression is rather complex and noninformative [59].

**Figure 2:**
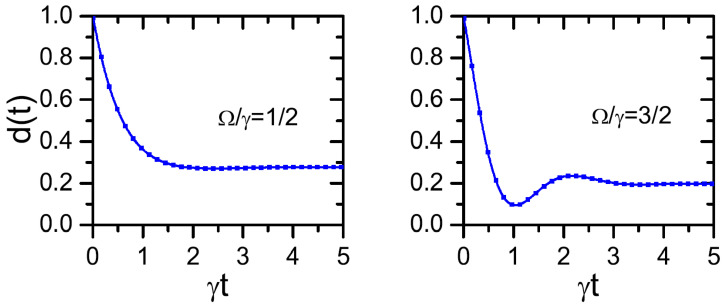
Decay of the trace distance d(t) (Equations (38) and (49)) corresponding to the model (45) with ϕ=γ for different values of the Hamiltonian frequency Ω/γ.

In this alternative situation, it is clear that $H_{e}$ induces intrinsic quantum coherent oscillations in the environment dynamics, which in turn may lead to oscillations in the trace distance (Equation (38)). In Figure 2, we plot the TD decay $d\left(t\right)=\left|4w(t)-1\right|/3$ taking ϕ=γ and for different values of Ω/γ. When Ω/γ<1, a monotonous decay is observed. Nevertheless, for Ω/γ>1, *revivals* in the TD are observed.

The CPF correlation in the deterministic scheme cannot be calculated in an analytical way. Nevertheless, given that the system dynamics is still controlled by the environment (self) transitions, it follows that $C_{pf}{\left(t,\tau \right)|}_{\overset{˘}{y}}\overset{d}{\neq }0.$ Thus, the dynamics become non-Markovian in both approaches (Ω/γ>1). Nevertheless, given that the environment is a casual bystander one, in the random scheme it is valid that $C_{pf}{\left(t,\tau \right)|}_{\overset{˘}{y}}\overset{r}{=}0$ (Equation (13)) for any value of Ω/γ. Consequently, in the same way as in the previous model (Equation (40)), the nonoperational and operational approaches give different results about the presence of environment-to-system backflows of information.

## 5. Summary and Conclusions

The interpretation of quantum memory effects in terms of an environment-to-system backflow of information is still under vivid debate. In this contribution, we presented a partial view of this problem by comparing how this concept is introduced and interpreted in nonoperational and operational approaches to quantum non-Markovianity.

Our main contribution is a comparison between both formalisms for different environment models. We considered casual bystander environments, which are characterized by a density matrix that does not depend on the system degrees of freedom. This class covers classical statistical mixtures of Markovian dynamics (Equation (14)), interactions with stochastic classical degrees of freedom (Equation (16)), and environmental quantum degrees of freedom (Equation (19)). In addition, we considered unitary system–environment models (Equation (22)).

As a nonoperational approach, we used the TD between two system states with different initial conditions. This formalism is characterized by the bound Equation (2). We have argued that, in general, it is not possible to predict if for a given model the TD presents or does not present revivals in its time behavior. This property is valid for all environmental models. In the case of casual bystander ones, the previous feature represents an obstacle for giving a consistent physical interpretation of any environment-to-system backflow of information defined as revivals in the TD (Equation (3)). In fact, for these dynamics, the system–environment correlations emerge due to the unidirectional dependence of the system dynamics in the state of the environment and its transitions. In particular, for stationary environments, it is not possible to know when the system–environment correlations lead to the presence or absence of backflows of information. The possibility of obtaining monotonous decay behaviors of the TD for unitary interaction models also represents an undesirable property because, in general, the environment state is modified by its interaction with the system.

As an operational approach, we used a CPF correlation (Equation (6)), which is defined by three consecutive system measurement processes. Both deterministic and random schemes were considered (with associated joint probabilities Equations (7) and (8)). In the case of casual bystander environments, the CPF correlation in the deterministic scheme does not vanish, while in the random scheme it vanishes identically for any chosen measurement observables (Equation (13)). Thus, in this approach, any casual bystander environment leads to a non-Markovian system dynamics but not any bidirectional information flow is detected. In the case of Hamiltonian models, in general, in both schemes the CPF correlation does not vanish, indicating non-Markovian system dynamics and the presence of bidirectional information flows (Equation (24)). An undesirable exception to this last property emerges when the system dynamics can equivalently be represented by a random unitary map (Equations (25) and (27)).

As a specific example, we considered a system coupled to an environment able to induce depolarizing dynamics (Equations (30a), (30b), (40) and (45)). We found that both approaches differ in the Markovian and non-Markovian regimes, as well in the presence or absence of environment-to-system backflows of information.

In general, both operational and nonoperational approaches to quantum non-Markovi- anity provide necessary and complementary points of view for defining and understanding memory effects in open quantum systems. The present results shed light on some conceptual differences and properties of these approaches. They may be useful for extending the application of these formalisms for the understanding of memory effects induced by structured or spatially extended environments.

## Data Availability

Not applicable.
